# Taking the temperature of the United States public regarding microbiome engineering

**DOI:** 10.3389/fpubh.2024.1477377

**Published:** 2024-12-11

**Authors:** Christopher Cummings, Kristen D. Landreville, Jennifer Kuzma

**Affiliations:** ^1^Genetic Engineering and Society Center, North Carolina State University, Raleigh, NC, United States; ^2^United States Army Corps of Engineers, Washington, DC, United States; ^3^School of Public and International Affairs, North Carolina State University, Raleigh, NC, United States

**Keywords:** microbiome engineering, built environment, survey, risk, public perception

## Abstract

This paper presents the first representative survey of U.S. adults’ opinions on microbiome engineering within the built environment, revealing public awareness, perceived benefits and risks, and attitudes toward genetically engineered microbiomes. Using data from a cross-sectional survey of 1,000 nationally representative U.S. residents over 18 years of age, we examined demographic and cultural factors influencing public sentiment. Results indicate that younger generations report higher knowledge levels, optimism, and perceived benefits of microbiome engineering, while older generations exhibit more caution and concern about risks. Political affiliation, education level, and trust in science also shape public attitudes, with Democrats, college-educated individuals, and those with higher trust in science more likely to view microbiome engineering positively. Notably, nearly half of respondents across demographic groups remain uncertain about the technology’s benefits and risks, and a majority of participants support government oversight to ensure ethical and responsible development. These insights provide a foundation for policymakers and researchers to foster informed public engagement and guide responsible innovation in microbiome engineering for built environments.

## Introduction

Microbiome engineering represents a frontier at the convergence of microbiology, genomics, and engineering, offering unprecedented opportunities to manipulate microbial communities in various environments (1). This field aims to enhance human health by selectively modifying the composition and function of these communities to achieve desired outcomes, such as mitigating disease risk or improving environmental conditions (2, 3). The built environment, encompassing all human-made spaces where people live, work, and play, serves as a critical focal point for research on the microbiome and interventions to improve public health (4). Here, the manipulation of microbial communities through microbiome engineering holds significant promise for public health, potentially transforming our living spaces into environments that actively contribute to health and well-being. For example, Clustered Regularly Interspaced Short Palindromic Repeats (CRISPR)-edited microbes could be used to degrade antimicrobial resistant genes in the harmful microbes that plague hospital sinks and contribute to the hundreds of thousands of hospital-acquired infections each year (5). Heating, Ventilation, and Air Conditioning (HVAC) systems could employ microbe-sensing technology to detect fungal mold spores in office buildings and then deploy beneficial microbes to displace the fungal contamination. This study specifically explores microbiome engineering within the built environment, focusing on how targeted microbial interventions can improve public health in spaces where people live, work, and interact. Examples include engineered microbiomes delivered as probiotics in home HVAC systems to colonize indoor spaces and thus replace harmful microbes such as mold or pathogens, or gene-edited microbes containing CRISPR systems targeted to degrade antimicrobial resistance genes in pathogens that colonize hospital sinks [e.g., (6)]. By examining public perceptions of these applications, this study addresses an emerging area within microbiome engineering with significant potential to impact health outcomes across diverse environments.

The United States, as a leader in microbiome research and biotechnology, provides a unique setting to understand public attitudes toward microbiome engineering in the built environment, especially as this field progresses toward practical applications. At this early stage, before genetically engineered microbiomes are deployed, insights into U.S. perspectives are crucial, as public opinion can significantly shape the governance and deployment of such technologies, not only domestically but as a model that may inform international efforts. Despite potential benefits, the introduction of microbiome engineering into the built environment raises complex societal and ethical implications (7). These range from considerations of privacy, informed consent, governance, and ownership to the equitable distribution of benefits and the management of risks. Moreover, the field’s rapid advancement underscores the urgent need for a comprehensive understanding of public perceptions. Public opinion can greatly influence the development, acceptance, and implementation of emerging technologies, making it a crucial factor in the successful integration of microbiome engineering into the built environment. Furthermore, the consideration and inclusion of public values early in the development of emerging technologies is a cornerstone of responsible research and innovation (RRI) (8). While applications of microbiome engineering are being considered but not yet deployed, it is a key time to assess public attitudes, hopes, and concerns about the technologies. As part of the team of a new National Science Foundation-funded Engineering Research Center on Precision Microbiome Engineering,^1^ one of the ways that we are working to employ a RRI approach in the early stages of the center’s technology development is through this assessment of U.S. public attitudes via a nation-wide survey.

Thus, this study provides a baseline assessment of public opinion on microbiome engineering within the built environment. This investigation is intended to serve as a primary touchstone to inform policymakers, researchers, and practitioners about the public’s stance on microbiome engineering, guiding responsible technology development and deployment as well as identification of strategies for public engagement, and anticipatory policy formation and oversight.

Existing research on public perceptions of biotechnology and microbiome engineering indicates a complex landscape influenced by factors such as knowledge, ethical considerations, cultural and demographic standpoints, risk and benefit distributions, trust in science, and governance (9–11). Studies highlight that while biotechnologies offer substantial benefits, concerns over safety, ethics, and the management of risks remain prevalent. Specifically, in the realm of microbiome engineering within built environments, there is a significant research gap on public opinions, despite the profound health implications of manipulating microbiomes where humans live and work (7, 12). Theoretical frameworks such as the deficit model, which suggests public skepticism of emerging technologies arises from a lack of knowledge (13), and more nuanced approaches like the contextual model, emphasize the importance of engaging public values to foster constructive dialogues about emerging technologies (14).

Additionally, the psychometric model (15) and the cultural cognition model (16) reveal that controllability and familiarity, cultural values, and worldviews significantly shape public perceptions and acceptance of scientific advancements. These insights underscore the necessity for anticipatory governance that integrates public values early in the development of technologies like microbiome engineering, aiming for alignment with societal needs and ethical standards, thereby addressing the critical need for focused research on public opinions toward microbiome engineering in built environments.

## Method

### Sampling procedure

A cross-sectional survey on public attitudes about microbiome engineering in the built environment was conducted using data collected from a nationally representative sample of 1,000 U.S. residents over 18 years of age, drawn from YouGov’s National Omnibus Panel during the first 2 weeks of December 2023 (17). 1,092 respondents were surveyed then matched down to a sample of 1,000 to produce the final dataset. The respondents were matched to a sampling frame on gender, age, race, and education. The sampling frame is a politically representative “modeled frame” of US adults, based upon the American Community Survey (ACS) public use microdata file, public voter file records, the 2020 Current Population Survey (CPS) Voting and Registration supplements, the 2020 National Election Pool (NEP) exit poll, and the 2020 CES surveys, including demographics and 2020 presidential vote. The results have an observed margin of error of ±3.38 percentage points. The National Omnibus is a compensated opt-in survey panel comprised of 1.8 million U.S. residents who have agreed to participate. Panel members are recruited through various methods to help ensure representativeness of the panel population, including web advertising, permission-based email contacts, partner-sponsored solicitations, telephone contacts using random digit dialing, and mail contacts using random address selection (18). While this study used an opt-in panel with demographic weighting to approximate a representative U.S. population, we acknowledge that some selection bias may still exist.

### Respondent characteristics

The average age of respondents was close to 49 years old (SD = 17.71 years), with a range from 19 to 88 years old. Using Pew Research Center’s generation categories, there were 149 Generation Z respondents (birth year 1997 and beyond), 251 Millennials (birth year 1981–1996), 248 Generation X (birth year 1965–1980), 311 Baby Boomers (birth year 1946–1964), and 41 Silent Generation (birth year 1945 or earlier). There was a fairly even distribution of men (*n* = 480) and women (*n* = 510), with 4 non-binary/genderqueer respondents, 5 transgender respondents, 2 respondents preferring not to say, and 1 respondent who reported “no label.”

About a third of respondents (*n* = 346) earned a four-year college degree or beyond, with the other two-thirds reporting no four-year college degree (*n* = 654). There were 267 lower income respondents (earned less than $30,000 per year), 443 middle income respondents (earned $30,000 to $99,999 per year), and 290 higher income respondents (earned more than $100,000 per year). Respondents reported being Independent and unaffiliated (*n* = 370), Democrat (*n* = 345), and Republican (*n* = 285).

### Measures

**Figure 1:**
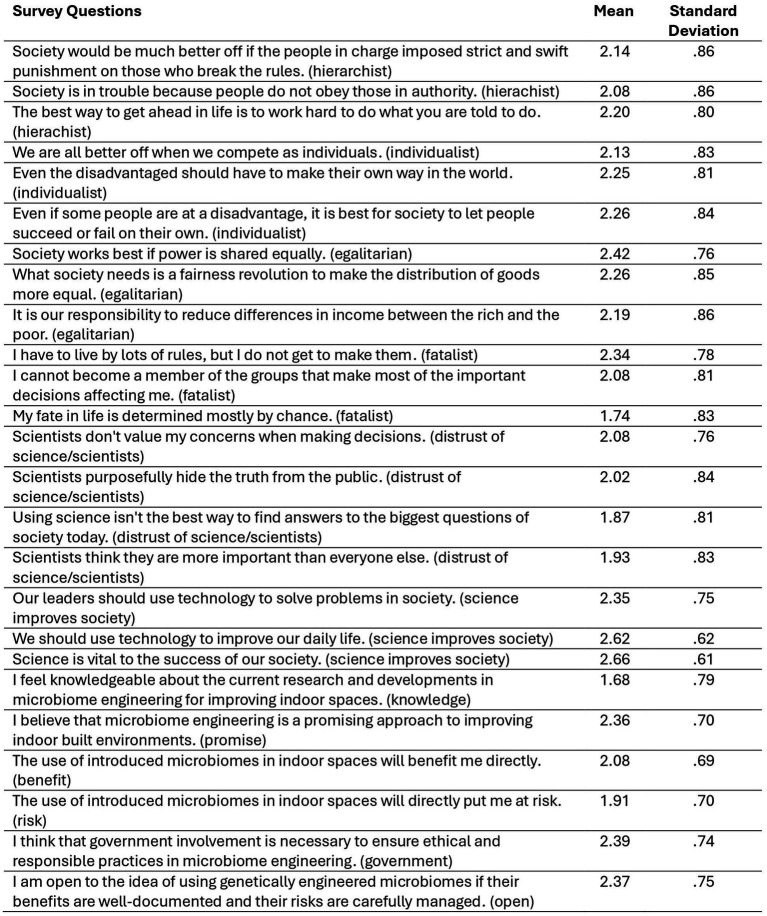
Descriptive statistics of cultural values scales, science and technology belief subscales, and key outcome variables. All questions used the response scale of 1 = disagree, 2 = unsure, 3 = agree.

Beyond using demographic and socioeconomic variables as predictor measures, the study used cultural theory’s four value-orientations. There were three items each to measure hierarchist, individualist, egalitarian, and fatalist orientations (19, 20). The three items for each orientation were averaged to create a composite score: hierarchist (*M* = 2.20, SD = 0.91, Cronbach’s *α* = 0.64), individualist (*M* = 2.27, SD = 0.88, Cronbach’s *α* = 0.70), egalitarian (*M* = 2.34, SD = 0.88, Cronbach’s *α* = 0.75), and fatalist (*M* = 2.06, SD = 0.89, Cronbach’s *α* = 0.42). See Figure 1 for the question wording and descriptive statistics. All questions used the response scale of 1 = disagree, 2 = unsure, 3 = agree.

Additionally, two subscales regarding science and technology beliefs were measured (21, 22). Four items comprised the ‘distrust in science/scientists’ subscale (*M* = 1.95, SD = 0.93, Cronbach’s *α* = 0.74) and three items comprised the ‘science improves society’ subscale (*M* = 2.67, SD = 0.66, Cronbach’s *α* = 0.64).

For analysis of the science and technology beliefs subscales and the cultural theory scales, a midpoint of 2 served as the differentiation between “more” and “less” categorization of a respondent’s composite score: hierarchist (“more” *n* = 535, “less” *n* = 336), individualist (“more” *n* = 562, “less” *n* = 293), egalitarian (“more” *n* = 611, “less” *n* = “274”), fatalist (“more” *n* = 436, “less” *n* = 371), distrust in science/scientists (“more” *n* = 406, “less” *n* = 454; note that “less” is phrased as “trust in science/scientists” in the figures), science improves society (“more” *n* = 780, “less” *n* = 108; note that “less” is phrased as “science does not improve society” in the figures). If a respondent’s composite score was at the midpoint, then they were not included in the results and figures for the sake of conserving space.

There were six distinct outcome measures used in the study to investigate (1) knowledge perceptions of microbiome engineering, (2) belief that microbiome engineering is a promising technology, (3) perceptions of benefit and (4) risk of the technology, (5) belief that government involvement is needed to oversee the technology, and (6) openness to using genetically engineered microbiomes. The full survey questions are listed at the bottom of Figure 1.

## Results

The results of this study provide insights into U.S. public perceptions of microbiome engineering within the built environment, highlighting the complex interplay between demographic factors, cultural values, and beliefs about science and technology. We uncover key trends across generations, political affiliations, and education levels. These findings offer a foundation for understanding public sentiment on this emerging field and underscore the need for targeted communication and policy strategies that address both enthusiasm and concerns among diverse population segments.

### Knowledge and promise of microbiome engineering

**Figure 2:**
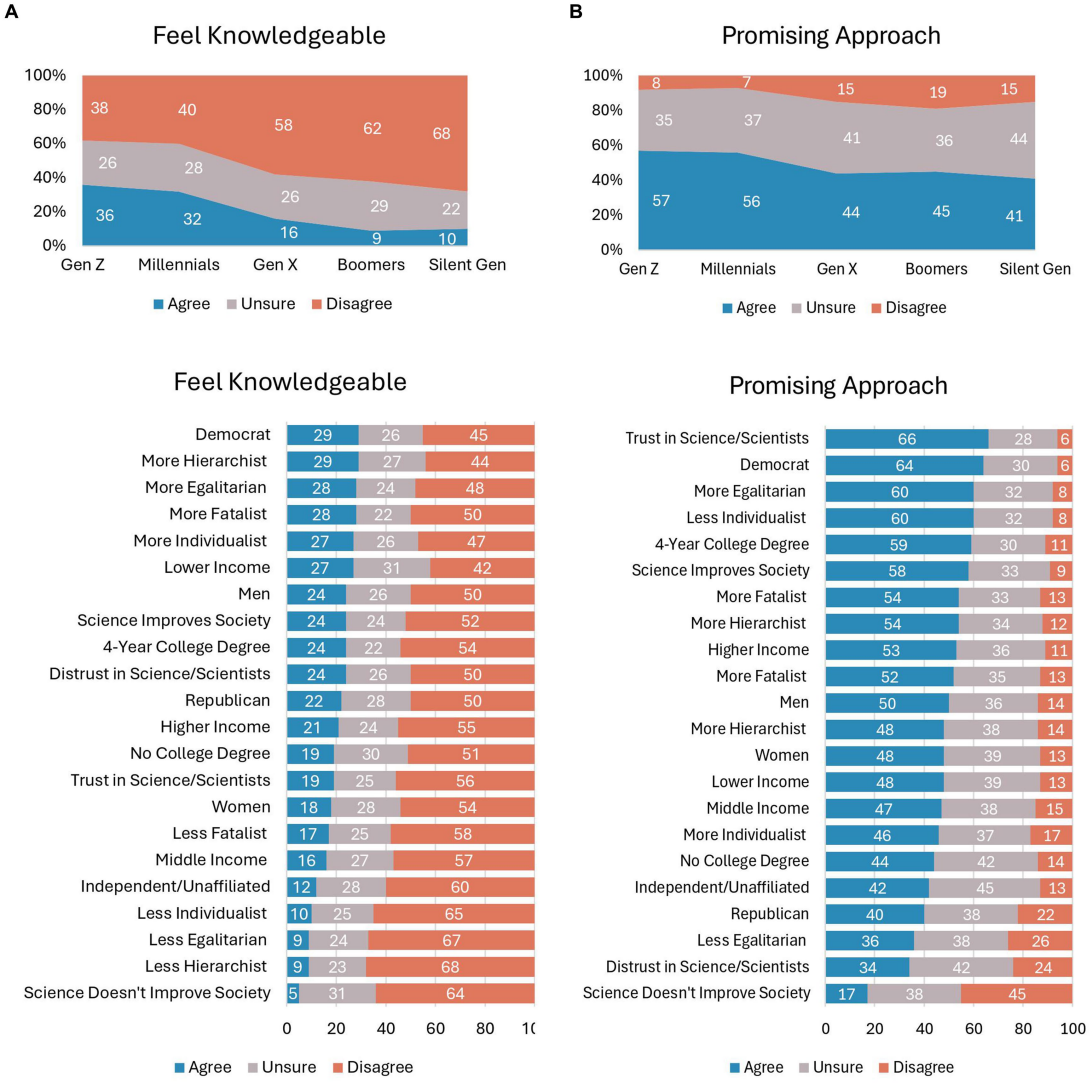
**(A)** Responses to the statement "I feel knowledgeable about the current research and developments in microbiome engineering for improving indoor spaces," according to respondents' demographic and psychographic characteristics. **(B)** Responses to the statement "I believe that microbiome engineering is a promising approach to improving indoor built environments," according to respondents' demographic and psychographic characteristics.

Younger generations feel more knowledgeable about microbiome engineering than older generations (see Figure 2A), *Χ*^2^(df = 8) = 81.47, *p* < 0.001, Cramer’s *V* = 0.202. About one-third of the Gen Z and Millennial generations feel knowledgeable, whereas only about 10% of Baby Boomer and Silent generations feel knowledgeable. Gen X falls in the middle, with 16% of that generation feeling knowledgeable. Going beyond age, Figure 2 shows that feeling knowledgeable about microbiome engineering varies by other demographics and social attitudes. In general, the respondents who feel most knowledgeable about microbiome engineering are Democrat (29% vs. 22% of Republicans and 12% of Independent/unaffiliated; *Χ*^2^(df = 4) = 36.16, *p* < 0.001, Cramer’s *V* = 0.190), men (24% vs. 18% of women; *Χ*^2^(df = 2) = 7.21, *p* < 0.05, Cramer’s *V* = 0.085), lower income (27% vs. 16% of middle income and 21% of higher income; *Χ*^2^(df = 4) = 18.06, *p* < 0.001, Cramer’s *V* = 0.095), and more hierarchist (29% vs. 9% less hierarchist; *Χ*^2^(df = 4) = 66.02, *p* < 0.001, Cramer’s *V* = 0.182), more egalitarian (28% vs. 9% less egalitarian; *Χ*^2^(df = 4) = 89.07, *p* < 0.001, Cramer’s *V* = 0.298), more fatalist (28% vs. 17% less fatalist; *Χ*^2^(df = 4) = 53.20, *p* < 0.001, Cramer’s *V* = 0.231), and more individualist (27% vs. 10% less individualist; *Χ*^2^(df = 4) = 44.31, *p* < 0.001, Cramer’s *V* = 0.210). Self-reported knowledge about microbiome engineering is lowest among respondents who say science does not improve society, which is at 5% (vs. 24% of respondents who say science improves society, *Χ*^2^(df = 8) = 35.63, *p* < 0.001, Cramer’s *V* = 0.133). In sum, there are low levels of knowledge among the public.

Even though knowledge levels about microbiome engineering are low, there are signs of hope among the public. Figure 2B shows that when considering respondents’ belief that microbiome engineering is a promising approach to improving the indoor built environment, statistically significant age differences emerge again, *Χ*^2^(df = 8) = 27.56, *p* < 0.001, Cramer’s *V* = 0.166. Majorities of the Gen Z (57%) and Millennial (56%) generations believe that microbiome engineering is a promising approach, but that is not the case with Gen X (44%), Baby Boomer (45%), and Silent (41%) generations. Other demographic groups who see promise in microbiome engineering are Democrats (64% vs. 40% of Republicans and 42% of Independent/unaffiliated; *Χ*^2^(df = 4) = 61.58, *p* < 0.001, Cramer’s *V* = 0.175) and individuals with college-education (59% vs. 44% of non-college-educated; *Χ*^2^(df = 2) = 18.36, *p* < 0.001, Cramer’s *V* = 0.135). Respondents with trust in science/scientists held strong belief that microbiome engineering is a promising approach compared to respondents who distrust in science/scientists (66% vs. 34%; *Χ*^2^(df = 4) = 130.85, *p* < 0.001, Cramer’s *V* = 0.256). Likewise, there are large differences in respondents who say science improves society vs. science does not improve society (58% vs. 17%; *Χ*^2^(df = 4) = 180.72, *p* < 0.001, Cramer’s *V* = 0.301). In short, while many respondents hold promise for microbiome engineering to improve the indoor built environment, there are consistently about one-third or more of respondents in all demographic groups who are unsure about the technology’s promise.

### Benefits and risks

**Figure 3:**
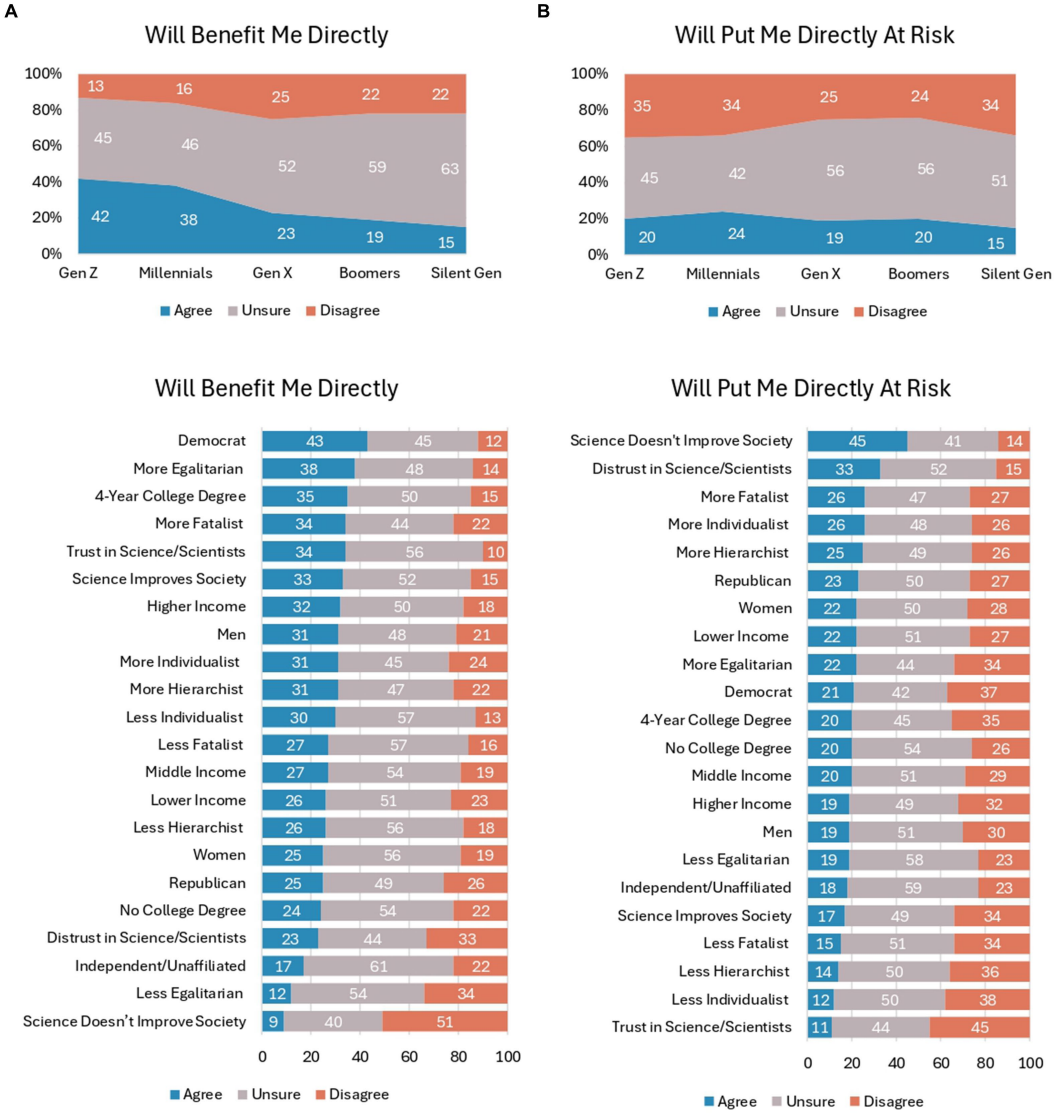
**(A)** Responses to the statement "The use of introduced microbiomes in indoor spaces will benefit me directly," according to respondents' demographic and psychographic characteristics. **(B)** Responses to the statement "The use of introduced microbiomes in indoor spaces will directly put me at risk," according to respondents' demographic and psychographic characteristics.

In addition to knowledge perceptions and belief that microbiome engineering is a promising approach, it is also important to understand how the public is weighing the direct benefits and risks of using introduced microbiomes in indoor spaces (see Figure 3A). Younger generations see more direct benefits (42% of Gen Z and 38% of Millennials) compared to older generations (23% of Gen X, 19% of Baby Boomers, and 15% of Silent), *Χ*^2^(df = 8) = 49.86, *p* < 0.001, Cramer’s *V* = 0.158. Differences are also found among political party affiliation (*Χ*^2^(df = 4) = 69.25, *p* < 0.001, Cramer’s *V* = 0.186) and education levels (*Χ*^2^(df = 2) = 16.27, *p* < 0.001, Cramer’s *V* = 0.128). Specifically, 43% of Democrats think using introduced microbiomes in indoor spaces will benefit them directly (vs. 25% of Republicans and 17% of Independent/unaffiliated) and 35% of college-educated respondents think the practice will benefit them (vs. 24% of non-college-educated respondents). Respondents who are more egalitarian (38% vs. 12% of less egalitarian; *Χ*^2^(df = 4) = 93.89, *p* < 0.001, Cramer’s *V* = 0.217) and more fatalist (34% vs. 27% of less fatalist; *Χ*^2^(df = 4) = 30.07, *p* < 0.001, Cramer’s *V* = 0.123) in their cultural values are also among the respondents who agreed the most that introducing microbiomes in indoor spaces will benefit them directly. About a third of respondents who trust in science/scientists and believe that science improves society agreed that the practice will benefit them as well, which were statistically significant differences from those who distrust science/scientists (*Χ*^2^(df = 4) = 82.77, *p* < 0.001, Cramer’s *V* = 0.203) and believe that science does not improve society (*Χ*^2^(df = 8) = 95.87, *p* < 0.001, Cramer’s *V* = 0.219).

Risk perceptions of new technologies are also critical to assess (see Figure 3B). Results show that there are generational differences in risk perceptions (*Χ*^2^(df = 8) = 17.50, *p* < 0.001, Cramer’s *V* = 0.094), with Gen Z and Millennials disagreeing significantly more than Gen X and Baby Boomers that introduced microbiomes in indoor spaces will put them at risk. Moving to other demographic and socioeconomic indicators, there are significant differences in disagreement about direct risks according to education (*Χ*^2^(df = 2) = 11.04, *p* < 0.001, Cramer’s *V* = 0.105) and political party (*Χ*^2^(df = 4) = 25.08, *p* < 0.001, Cramer’s *V* = 0.112). In particular, college degree earners are more likely to disagree that there are direct risks (35% vs. 26% of non-college-degree earners), and Democrats are more likely than Republicans and Independent/unaffiliated to disagree there are direct risks (37% vs. 27% and 23%, respectively). There are statistically significant differences among respondents according to their science and technology beliefs: 45% of respondents who think science does not improve society are concerned about introduced microbiomes in indoor spaces putting them directly at risk, while only 17% of respondents who think science improves society are concerned (*Χ*^2^(df = 4) = 76.67, *p* < 0.001, Cramer’s *V* = 0.196). Likewise, 33% of respondents who report distrust in science/scientists are concerned vs. 11% of respondents who report trust in science/scientists (*Χ*^2^(df = 4) = 131.47, *p* < 0.001, Cramer’s *V* = 0.256).

Overall, when comparing benefit vs. risk perceptions, respondents exhibit slightly more belief that the use of introduced microbiomes in indoor spaces will directly benefit them than put them at risk. However, it is important to emphasize the very large number of respondents who are unsure what to believe about the benefits and risks, as evidenced by the nearly 50% of respondents among various demographic, socioeconomic, cultural value beliefs, and science and technology beliefs who report being unsure.

### Government involvement and openness to genetically engineered microbiomes

**Figure 4:**
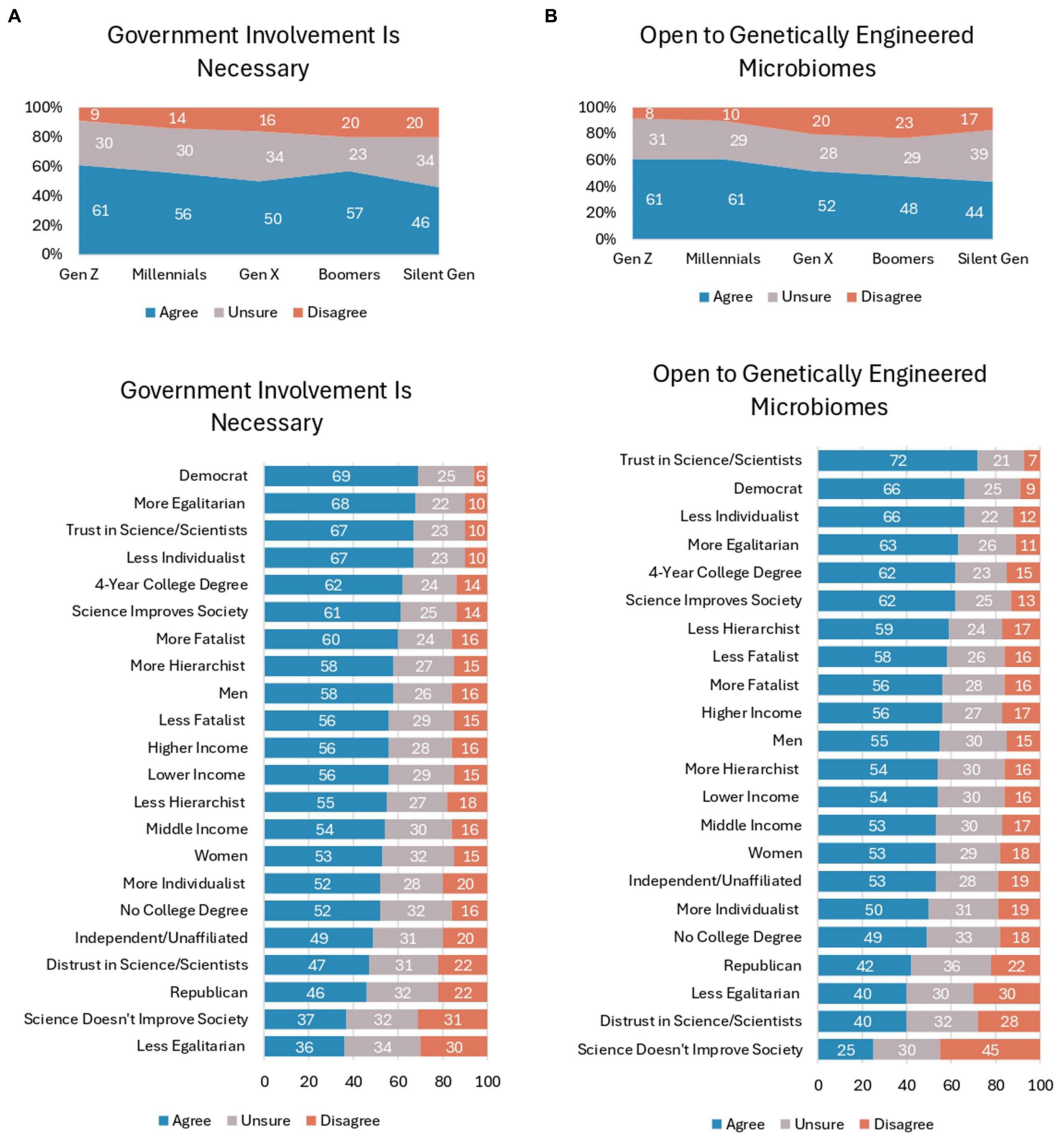
**(A)** Responses to the statement "I think that government involvement is necessary to ensure ethical and responsible practices in microbiome engineering," according to respondents' demographic and psychographic characteristics. **(B)** Responses to the statement "I am open to the idea of using genetically engineered microbiomes if their benefits are well-documented and their risks are carefully managed," according to respondents' demographic and psychographic characteristics.

With so much uncertainty among the public about the benefits and risks microbiome engineering for the built environment, it is not surprising that majorities of nearly all generations want government involvement to ensure ethical and responsible practices for the developing technology (see Figure 4). Specifically, Gen Z has the highest support for government involvement (61%), followed by Baby Boomers (57%), Millennials (56%), Gen X (50%), and the Silent Generation (46%), with the only statistically significant difference occurring between Gen Z and Gen X (*Χ*^2^(df = 8) = 17.75, *p* < 0.001, Cramer’s *V* = 0.094). The group with the most support for government involvement are Democrats (69%), and their support is significantly higher than Republicans (46%) and Independents/unaffiliated (49%), *Χ*^2^(df = 4) = 54.55, *p* < 0.001, Cramer’s *V* = 0.165. College-educated respondents show significantly higher levels of support for government involvement as well (62% vs. 52% of non-college-educated; *Χ*^2^(df = 2) = 9.19, *p* < 0.05, Cramer’s *V* = 0.096). There is also more support for government involvement among respondents with more egalitarian values (68% vs. 36% of less egalitarian; *Χ*^2^(df = 4) = 45.96, *p* < 0.001, Cramer’s *V* = 0.152) and less individualist values (67% vs. 52% of more individualist; *Χ*^2^(df = 8) = 32.96, *p* < 0.001, Cramer’s *V* = 0.128). Interestingly, among respondents who trust in science/scientists, there is still strong support for government involvement to ensure ethical and responsible practices (67% vs. 47% of respondents who distrust in science/scientists; *Χ*^2^(df = 4) = 58.39, *p* < 0.001, Cramer’s *V* = 0.171). The lowest levels of support for government involvement exist among respondents who think science does not improve society (37% vs. 61% who science improves society; *Χ*^2^(df = 4) = 60.85, *p* < 0.001, Cramer’s *V* = 0.174).

In looking ahead to the public’s openness to using genetically engineered microbiomes in indoor spaces, younger generations are significantly more inclined to be open to the technology compared to older generations (*Χ*^2^(df = 8) = 32.96, *p* < 0.001, Cramer’s *V* = 0.128), as both Gen Z and Millennials show 61% openness, which is more than Gen X (52%), Baby Boomers (48%), and the Silent Generation (44%). There are statistically significant gaps in openness to genetically engineered microbiomes according to political identification (*Χ*^2^(df = 4) = 42.45, *p* < 0.001, Cramer’s *V* = 0.146) and education levels (*Χ*^2^(df = 2) = 14.31, *p* < 0.001, Cramer’s *V* = 0.120). Democrats (66% vs. 42% of Republicans and 53% of Independents/unaffiliated) and college-educated respondents (62% vs. 49% of non-college-educated) are among the most likely to be open. Cultural values continue to contribute to significant differences in views on microbiome engineering, with less individualist (66% vs. 50% more individualist; *Χ*^2^(df = 4) = 27.82, *p* < 0.001, Cramer’s *V* = 0.118) and more egalitarian (63% vs. 40% less egalitarian; *Χ*^2^(df = 4) = 82.51, *p* < 0.001, Cramer’s *V* = 0.203) respondents showing high levels of openness. Respondents with trust in science/scientists exhibit the highest levels of openness to genetically engineered microbiomes (72% vs. 40% of respondents with distrust in science/scientists; *Χ*^2^(df = 4) = 138.44, *p* < 0.001, Cramer’s *V* = 0.372). Additionally, 62% of respondents who believe science improves society are open vs. 25% of respondents who believe science does not improve society (*Χ*^2^(df = 4) = 131.47, *p* < 0.001, Cramer’s *V* = 0.256).

In sum, most respondents are open to the idea of using genetically engineered microbiomes if the benefits are well-documented and the risks are carefully managed. Even still, there are about a quarter to a third of respondents in various groups who are uncertain about genetically engineered microbiomes. Fairly low numbers of respondents among various groups disagree that government involvement is necessary and disagree that they are open to genetically engineered microbiomes.

## Discussion

This study provides an initial exploration of U.S. public perceptions of microbiome engineering within the built environment, uncovering notable generational, political, and educational differences in knowledge, perceived benefits and risks, and support for government oversight. The findings reveal a blend of optimism and caution, highlighting both enthusiasm for the potential benefits of microbiome engineering and significant uncertainty due to limited familiarity with its specific applications. This suggests that targeted engagement strategies may be essential to build public awareness and understanding of this emerging field.

The results offer a valuable snapshot of public sentiment, underscoring the importance of transparent communication to clarify both the capabilities and limitations of microbiome technologies as they advance. Public support for microbiome engineering is intertwined with perceptions of its potential to enhance indoor environments, but uncertainties around risks highlight a need for improved public discourse and responsible development as the field matures. By addressing these perceptions and questions early in the field’s development, stakeholders can better align technological advancements with public values, fostering a responsible pathway for microbiome engineering within built environments. The results of this study highlight the nuanced landscape of public perceptions surrounding microbiome engineering in the built environment, including significant generational differences. Younger generations, such as Gen Z and Millennials, generally report higher optimism toward microbiome engineering—57 and 56%, respectively, view it as promising—potentially reflecting their familiarity with biotechnology and digital advancements. In contrast, older generations like Baby Boomers (45%) and the Silent Generation (41%) demonstrate more cautious views, which may relate to lower exposure to such technologies and a more conservative perspective. This suggests that tailored information approaches may be beneficial to support independent attitude formation and informed decision-making across generations, enabling individuals to consider microbiome technology adoption in ways that align with their knowledge levels and concerns. For example, younger audiences might benefit from practical information on applications and risk management, while older audiences may find foundational and context-setting information more helpful in building informed perspectives.

Furthermore, the results suggest a substantial demand for government oversight. The public’s call for regulatory involvement underscores a collective desire for assurances that the development and implementation of microbiome technologies will adhere to ethical standards and responsible practices. This is not only a plea for safeguarding against risks but also an indication of the public’s readiness to accept and adopt genetically engineered microbiomes, similar to other biotechnologies, provided their benefits are convincingly demonstrated, potential risks are managed, and that ethical and social implications are judiciously considered (23). This aligns with the principles of the RRI framework which emphasizes the integration of public values, societal needs, and ethical considerations from the early stages of technological development. By leveraging insights from this survey, policymakers and researchers are better positioned to anticipate and navigate the societal impacts and public receptivity toward microbiome engineering. In practice, this means cultivating a research and development ecosystem that not only pushes the boundaries of scientific innovation but also remains deeply attuned to the ethical, social, and cultural implications of such advancements. Effective public engagement strategies that invite community participation in the decision-making processes are crucial for building trust and fostering a sense of ownership among stakeholders, thereby ensuring that the development of microbiome technologies does not just proceed with technical and scientific rigor but also with societal consent and foresight. Thus, this survey not only maps the current landscape of public opinion but also acts as a directive for future actions—calling for a proactive approach to integrate public insights into the scientific discourse, which will strengthen the ethical development of technologies as well as the governance frameworks guiding microbiome engineering deployment.

Future research could also seek to identify factors influencing individuals’ intentions either to adopt or purposefully avoid microbiome engineering technologies, examining variables such as perceived benefits, risks, trust in science, and cultural or generational values. Qualitative research exploring the underlying motivations and concerns across different demographics would offer a richer understanding of these factors, while international comparisons could reveal cultural variations that inform a global approach to public engagement and policy. Together, these approaches ensure that the evolution of microbiome technologies in the built environment is responsibly aligned with both scientific possibilities and public expectations, facilitating a balanced advancement that is technically feasible and socially endorsed.

## Data Availability

The datasets presented in this article are not readily available because data may be available upon request. Requests to access the datasets should be directed to Christopher Cummings clcummin@ncsu.edu.

## Footnotes

^1^ https://premier-microbiome.org

## References

[1] Sonnenburg J. Microbiome engineering. Nature. (2015) 518:S10. doi: 10.1038/518S10a 25715274

[2] Bai X Huang Z Duraj-Thatte AM Ebert MP Zhang F Burgermeister E. Engineering the gut microbiome. Nat Rev Bioeng. (2023) 1:665–79. doi: 10.1038/s44222-023-00072-2

[3] Jansson JK McClure R Egbert RG. Soil microbiome engineering for sustainability in a changing environment. Nat Biotechnol. (2023) 41:1716–28. doi: 10.1038/s41587-023-01932-3, PMID: 37903921

[4] NASEM. Microbiomes of the built environment: a research agenda for indoor microbiology, human health, and buildings. Washington, DC: National Academies Press (2017). 29035489

[5] CDC. (2023). HAI pathogens and antimicrobial resistance report, 2018-2021. Available at: https://www.cdc.gov/nhsn/hai-report/data-tables-adult/index.html (accessed June 3, 2024).

[6] Tao S Chen H Li N Liang W. The application of the CRISPR-Cas system in antibiotic resistance. Infect Drug Resist. (2022) 15:4155–68. doi: 10.2147/IDR.S370869, PMID: 35942309 PMC9356603

[7] Hardwick A Cummings C Graves J Jr Kuzma J. Can societal and ethical implications of precision microbiome engineering be applied to the built environment? A systematic review of the literature. Environ Syst Decis. (2024) 44:215–38. doi: 10.1007/s10669-024-09965-y

[8] Stilgoe J Owen R Macnaghten P. Developing a framework for responsible innovation. Res Policy. (2013) 42:1568–80. doi: 10.1016/j.respol.2013.05.008

[9] Bearth A Kaptan G Kessler SH. Genome-edited versus genetically-modified tomatoes: an experiment on people’s perceptions and acceptance of food biotechnology in the UK and Switzerland. Agric Hum Values. (2022) 39:1117–31. doi: 10.1007/s10460-022-10311-8

[10] De Witt A Osseweijer P Pierce R. Understanding public perceptions of biotechnology through the “integrative worldview framework”. Public Underst Sci. (2017) 26:70–88. doi: 10.1177/0963662515592364, PMID: 26142147

[11] Robinson JM Redvers N Camargo A Bosch CA Breed MF Brenner LA. Twenty important research questions in microbial exposure and social equity. mSystems. (2022) 7:e01240–21. doi: 10.1128/msystems.01240-21 35089060 PMC8725600

[12] Li S Yang Z Hu D Cao L He Q. Understanding buildingoccupant-microbiome interactions toward healthy built environments: a review. Front Environ Sci Eng. (2021) 15:65. doi: 10.1007/s11783-020-1357-3, PMID: 33145119 PMC7596174

[13] Liang T De Kok TM Abee T Schutyser MA. Precision microbiome engineering: opportunities and limitations. Front Microbiol. (2019) 10:1–8. doi: 10.3389/fmicb.2019.00001, PMID: 30728808 PMC6351783

[14] Cummings C Peters DJ. Who trusts in gene-editing foods? An analysis of a representative survey study predicting willingness to eat- and purposeful avoidance of gene edited foods in the United States. Front Food Sci Technol. (2022) 2:858277. doi: 10.3389/frfst.2022.858277

[15] Slovic P. Perception of risk: reflections on the psychometric paradigm In: Krimsky S Godling D, editors. Social theories of risk. Wesport, CT: Praeger (1992). 117–52.

[16] Kahan DM Jenkins-Smith H Braman D. Cultural cognition of scientific consensus. J Risk Res. (2011) 14:147–74. doi: 10.1080/13669877.2010.511246

[17] Cummings C. (2018). “Cross-sectional design,” in The SAGE encyclopedia of communication research. Editor Allen M. (Thousand Oaks: SAGE Publications), 315–317.

[18] YouGov. Survey procedures: Information related to the treatment of human subjects. Redwood City, CA: Scientific Research, YouGov (2020).

[19] Ripberger JT Gupta K Silva CL Jenkins-Smith HC. Cultural theory and the measurement of deep Core beliefs within the advocacy coalition framework. Policy Stud J. (2014) 42:509–27. doi: 10.1111/psj.12074

[20] Swedlow B Johnson BB Mayorga M. Cultural theory and cultural cognition theory survey measures: confirmatory factoring and predictive validity of factor scores for judged risk. SSRN Electron J. (2019). doi: 10.2139/ssrn.3345279

[21] Cummings CL Chuah ASF Ho SS. Protection motivation and communication through Nanofood labels: improving predictive capabilities of attitudes and purchase intentions toward Nanofoods. Sci Technol Hum Values. (2018) 43:888–916. doi: 10.1177/0162243917753991

[22] Rosenthal S Cummings CL. Influence of rapid COVID-19 vaccine development on vaccine hesitancy. Vaccine. (2021) 39:7625–32. doi: 10.1016/j.vaccine.2021.11.014, PMID: 34802786 PMC8590511

[23] Trump B Cummings C Klasa K Galaitsi S Linkov I. Governing biotechnology to provide safety and security and address ethical, legal, and social implications. Front Genet. (2023) 13:1052371. doi: 10.3389/fgene.2022.1052371, PMID: 36712887 PMC9873990

